# Echinocandins Accelerate Particle Transport Velocity in the Murine Tracheal Epithelium: Dependency on Intracellular Ca^2+^ Stores

**DOI:** 10.1128/AAC.00669-21

**Published:** 2021-10-18

**Authors:** Sabrina Müller, Maximilian Carl Droll, Christian Koch, Sebastian Weiterer, Markus A. Weigand, Michael Sander, Michael Henrich

**Affiliations:** a Department of Anaesthesiology, Intensive Care Medicine and Pain Therapy, University Hospital of Giessen and Marburg, Giessen, Germany; b Department of Anaesthesiology and Intensive Care Medicine, Bundeswehr Central Hospital Koblenz, Koblenz, Germany; c Department of Anaesthesiology and Intensive Care Medicine, Lukas Hospital Rheinland Clinic Neuss GmbH, Neuss, Germany; d Department of Anaesthesiology and Intensive Care Medicine, Heidelberg University Hospital, Heidelberg, Germany; e Department of Anaesthesiology, Intensive Care Medicine, Emergency Medicine, Vidia St. Vincentius-Clinic Karlsruhe gAG, Karlsruhe, Germany

**Keywords:** particle transport velocity, echinocandins, mucociliary clearance, intracellular calcium, antifungal agents

## Abstract

The mucociliary clearance of lower airways is modulated by different physiologic stimuli and also by pathophysiologic agents like polluting substances or pharmaceutical molecules. In the present investigation, we measured the particle transport velocity (PTV) of mouse tracheae as a surrogate for mucociliary clearance. In mouse tracheal preparations, we detected a sustained increase in the PTV under the application of the echinocandins caspofungin, anidulafungin, and micafungin. In further experiments, we observed the effects of echinocandins on the PTV were dependent on intracellular Ca^2+^ homeostasis. In Ca^2+^-free buffer solutions, the amplitude of the echinocandin-evoked rise in the PTV was significantly reduced relative to that in the experiments in Ca^2+^-containing solutions. Depletion of intracellular Ca^2+^ stores of the endoplasmic reticulum (ER) by caffeine completely prevented an increase in the PTV with subsequent caspofungin applications. Mitochondrial Ca^2+^ stores seemed to be unaffected by echinocandin treatment. We also observed no altered generation of reactive oxygen species under the application of echinocandins as probable mediators of the PTV. Consequently, the observed echinocandin effects on the PTV depend upon the Ca^2+^ influx and Ca^2+^ contents of the ER. We assume that all three echinocandins act intracellularly on ER Ca^2+^ stores to activate Ca^2+^-dependent signal transduction cascades, enhancing the PTV.

## INTRODUCTION

Mucociliary clearance of the lower airways is a pivotal mechanism to prevent deposition or colonization of the lungs by potential pathogens (1). This clearance function depends upon the metachronal continuous beating of cilia located on the apical membranes of tracheal epithelial cells (2, 3). The cilia immerge into the soluble phase of the mucus layer, and their continuous movement propels the mucus forward toward the gastrointestinal tract (4, 5), thereby removing particles trapped in the upper mucus layer from the lower airways (6). This particle transportation can be accelerated by increasing the ciliary beat frequency (CBF), which leads to more effective mucus flow (7–10). Here, the importance of proper cilia coordination of ciliary beating has to be accentuated, while in patients suffering from ciliopathies, like primary cilia dykinesia (PCD), the ciliary dysfunction leads to an ineffective mucociliary clearance, resulting in recurrent or chronic bacterial infections (11, 12). Mucociliary clearance is influenced by multiple physiological signal cascades, including humoral, neural, mechanical, and immunologic processes (13–17). The invocation of many control mechanisms leads to enhanced CBF and, consequently, increased particle transport velocity (PTV), with improved rates of lower airway clearance. However, the CBF can also be inhibited by several factors, such as a drop in temperature, acidosis, reactive oxygen species (ROS), or polluting agents. All these determinants lead to a reduced PTV and subsequent ineffective mucociliary clearance (5, 18–21).

Many pharmacological agents have been described to date as direct or indirect modulators of mucociliary clearance (22–24). These pharmacological agents reach ciliary-bearing tracheal epithelial cells via diffusion from blood vessels or by inhalation in order to interfere with them (25, 26). They may attain concentrations that are sufficient enough to alter CBF and, furthermore, for many pharmacological agents, e.g., beta-sympathomimetics or purines, research has elucidated how they influence cilia-bearing cells (27, 28). Most pharmacological substances act primarily on membrane-bound receptors or ion channels (29–32). However, they are also able to directly alter intracellular signal cascades or modulate cellular energy metabolism, leading to an altered turnover of ATP, which is a main activator and substrate of the cilia motion apparatus (32). Especially in critically ill patients or immunocompromised individuals, the mucociliary function plays a key role in reducing the risk of bacterial colonization of the lower airways, which would result in severe infections (33–35). Besides the risk for bacterial infection, severe pulmonary fungal infections, e.g., by *Candida* or Aspergillus, represent significant hazards that trigger increased mortality in critically ill or in immunocompromised patients (36, 37). In these patients, the use of effective antifungal therapies is crucial to reduce the fungal load and lessen the risk for organ failure and mortality (38–40).

In recent years, new strategies for the therapy of systemic fungal infections have been developed, including the administration of echinocandins, a new class of antimycotic drugs that are effective against invasive fungal infections, especially of diverse *Candida* strains (41–43). This class of antimycotic drugs mainly includes caspofungin, anidulafungin, and micafungin, which can reach high concentrations in the airways, since they are also suitable for direct bronchial administration via a nebulizer (44). However, so far, it is not known whether echinocandins can influence the mucociliary clearance or intracellular signal cascades of ciliary-bearing epithelial cells. In the present study, we investigated the PTV as an indicator of the mucociliary function of the lower airways under exposure to all three clinical established echinocandins in organ preparations of mouse tracheae. We elucidated the possible signal cascades involved in how echinocandins influence mouse tracheal epithelial function by pharmacological intervention. We separately inhibited receptors, kinases, and proteins of the concerned main signal cascades known to stimulate the PTV by administering pharmacological inhibitors. We also investigated whether echinocandins induce the generation of ROS in tracheal preparations as a hazard of many cell functions. Classically, ROS generation mainly occurs as a by-product of mitochondrial oxidative phosphorylation. ROS are highly reactive molecules that can oxidize other molecules of various cell signaling pathways, while the liberation of ROS acts like an alarm system of the cell during hypoxia, inflammation, or contact with pathogens (45–47).

The amount of ROS liberation plays a crucial role in cell homeostasis. Moderate ROS liberation leads to cell adaption to the stressor, while larger amounts of ROS could cause cell damage or even induce cell death (48). We performed ROS measurements to investigate if caspofungin induces ROS liberation, indicating cell stress or cell damage.

## RESULTS

**FIG 1:**
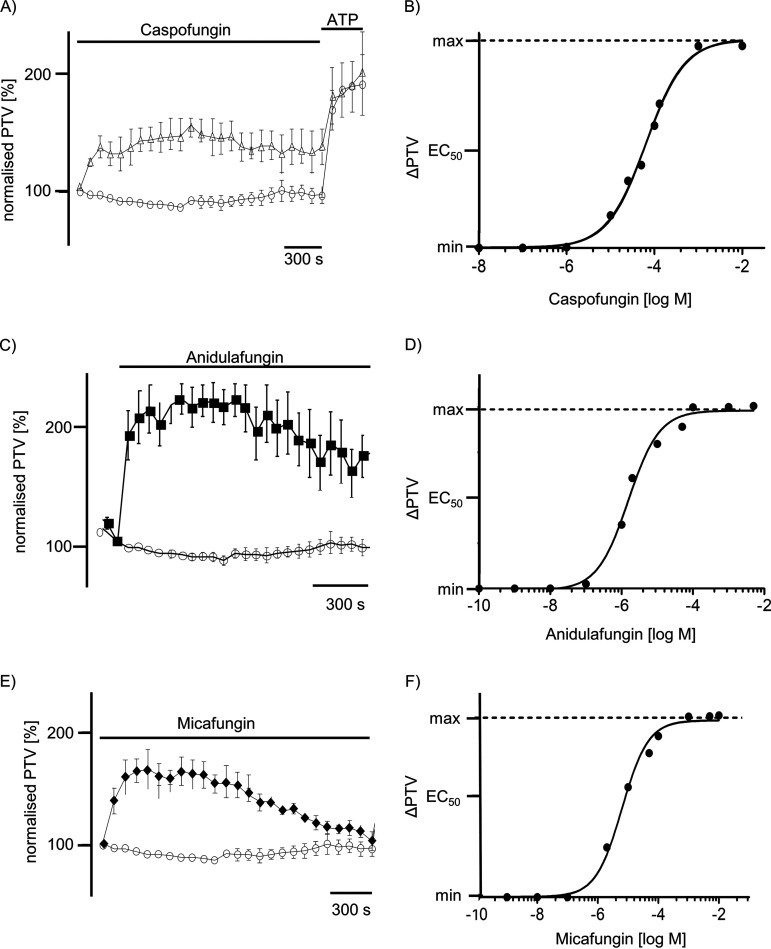
Echinocandins elicited an increase in the PTV in mice trachea. (A, C, and E) Caspofungin (60 μM), anidulafungin (44 μM), or micafungin (50 μM) rapidly induced a sustained elevation in the PTV that reached up to 200% of the basal PTV of control tracheae. Only experiments responding to ATP (100 μM) at the end of the exposure period were included for further statistical evaluation. (B) A dose-response curve presents the effects of caspofungin on the PTV. Using the Hill equation, EC_50_ was estimated at 13.4 μM. (C) Anidulafungin rapidly triggered an increase in the PTV, reaching more than 200% of the basal PTV under control conditions. (D) A dose-response curve presents the concentration-dependent effect of anidulafungin on the PTV following a Hill equation EC_50_ value of 6.5 μM. (E) Micafungin induced an increase in the PTV that seemed rather transient compared to that of caspofungin or anidulafungin; the maximum response reached more than 170% of the initial basal PTV. (F) The micafungin response occurred in a concentration-dependent manner. The Hill equation EC_50_ value for micafungin was estimated as 7.4 μM. In all panels, the PTV of the initial recording time point was normalized to 100%. Recordings show mean ± standard error of the mean values for individual time points. o, control; Δ, caspofungin; ■, anidulafungin; ♦, micafungin; horizontal bars in experimental recordings present exposure periods of defined pharmacological agents; time scale bars are present in all individual panels.

The application of either caspofungin, anidulafungin, or micafungin significantly increased the PTV of the mouse tracheal epithelium in a concentration-dependent manner (Fig. 1A to F). Under control conditions without the administration of echinocandins, the PTV experienced no alterations during the observation period (Fig. 1A, C, and E). All three tested echinocandins evoked a prolonged elevation of the PTV that declined only slightly with long-term exposure; still, the tracheae responded to ATP as a vitality control at the end of all individual experiments, which is presented exemplarily for all tests in Fig. 1A. An acceleration of the PTV following drug application became evident after a brief delay, which then rapidly reached a sustained plateau. The response to echinocandins occurred in a dose-dependent manner, following a Hill equation with 50% effective concentration (EC_50_) values of 13.4 μM for caspofungin (Fig. 1B), 6.5 μM for anidulafungin (Fig. 1D), and 7.4 μM for micafungin (Fig. 1F), respectively.

**FIG 2:**
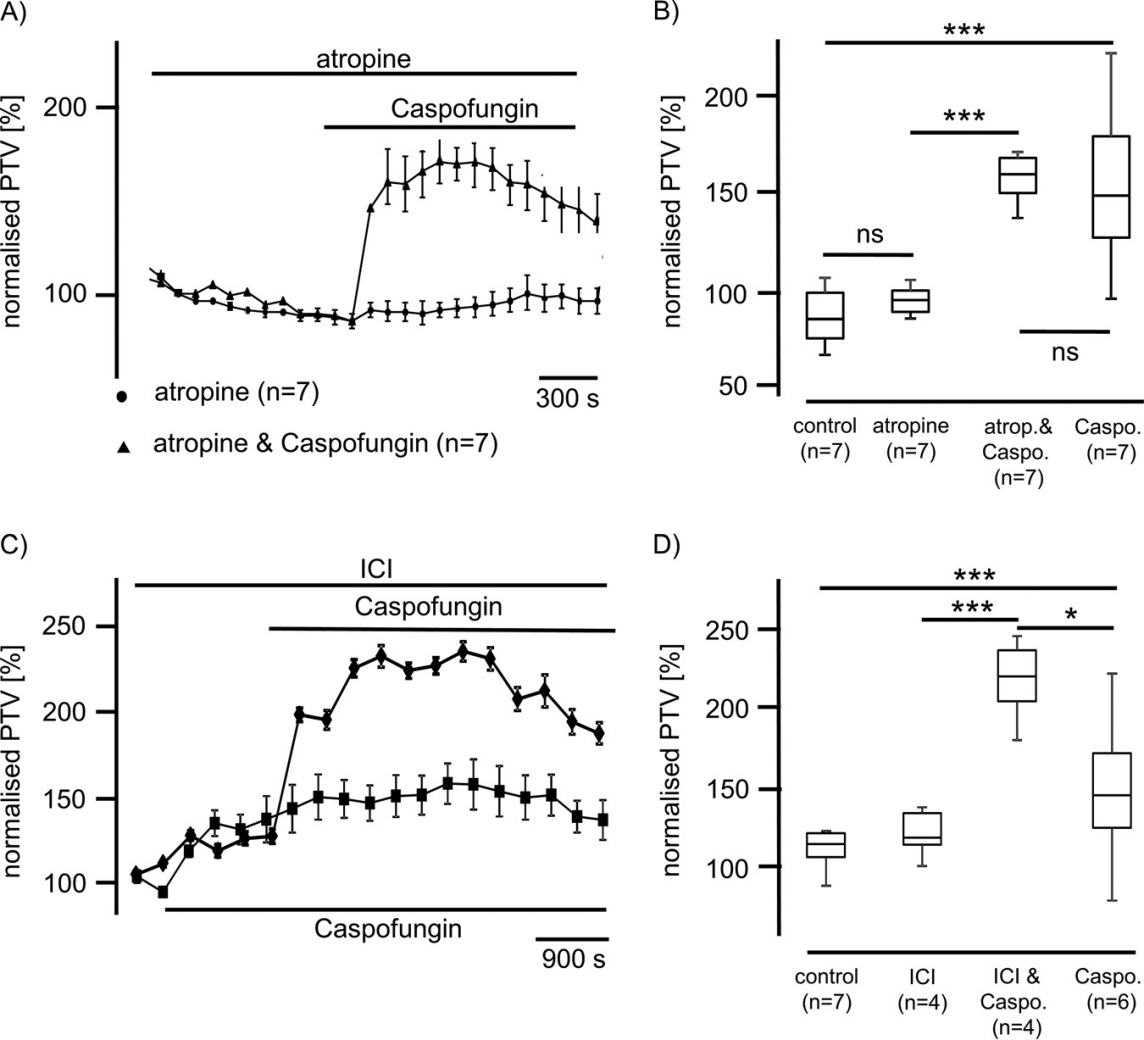
Caspofungin effects are independent of muscarinic or adrenergic receptor activation. (A) Atropine (10 μM) did not influence the basal PTV when applied alone or prior to caspofungin. Caspofungin (60 μM) application in the presence of atropine immediately induced a sustained increase in the PTV (●, atropine; ▴, atropine and caspofungin). (B) Atropine did not influence the basal PTV (paired Student's *t* test). Caspofungin application in the presence of atropine induced a significant rise in the PTV, which was not different from that seen in experiments when only caspofungin was applied. (C) The nonselective inhibitor of the adrenergic receptor ICI (10 μM) also induced a rise in the PTV, and application of caspofungin in the presence of ICI further evoked a sustained rise in the PTV that was higher than that triggered by caspofungin alone (■, caspofungin; ♦, ICI and caspofungin). (D) Exposure to caspofungin in the presence of ICI induced a significant rise in the PTV that was also different from that measured under caspofungin application alone. (atrop., atropine; caspo., caspofungin; horizontal bars in experimental recordings present exposure periods of defined pharmacological agents; *n*, number of individual experiments; ns, not significant; time scale bars in all individual panels; *, *P* < 0.05; ***, *P* < 0.001; Mann-Whitney U test).

Different signal pathways are described to alter the mouse tracheal PTV, also including membrane-bound receptors. Due to similar characteristics for all three echinocandins, we only show data for caspofungin concerning the investigation of membrane-bound receptors. In subsequent experiments, we added atropine (10 μM) to inhibit muscarinic receptors prior to caspofungin application. We observed no intrinsic effect of atropine on the PTV (Fig. 2A). The following application of caspofungin, still in the presence of atropine, evoked a significant increase of the PTV to 163% ± 12% (*n* = 7; *P* < 0.001) relative to control therapy. Atropine did not reduce the effect of caspofungin on the PTV (Fig. 2B).

ICI-118,511 (10 μM), a nonselective inhibitor of adrenergic beta-receptors, induced a significant increase in the PTV to 137%. However, the subsequent administration of caspofungin still in the presence of ICI further increased the PTV to 240% ± 11% (*n* = 4). This increase in the PTV was significantly greater (245%) than that observed during sole ICI exposure (137%) or exposure to caspofungin only (Fig. 2C and D).

**FIG 3:**
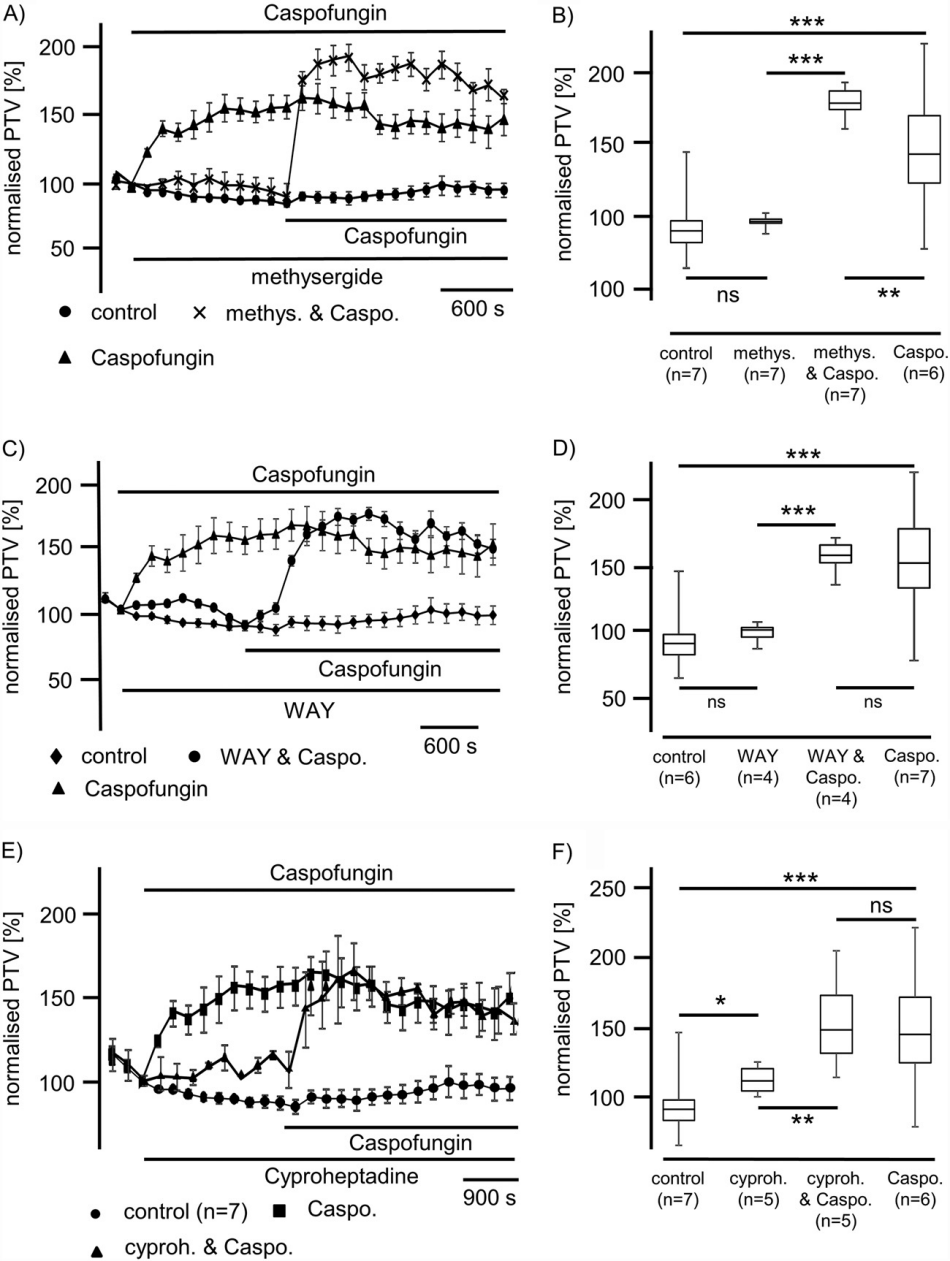
Caspofungin effects are evoked independently from serotonin receptors. (A) A nonselective inhibitor of serotonin receptors 5-HT_1_ and 5-HT_2_, methysergide (100 μM), did not alter the PTV. Caspofungin was applied in the presence of methysergide and immediately induced a rise in the PTV to a stable plateau above the PTV level that was evoked by caspofungin alone (●, control; ▴, caspofungin; x, methysergide and caspofungin). (B) Methysergide did not influence the PTV relative to control conditions (*P* = 0.03, Student’s paired *t* test). Caspofungin induced a significant increase in the PTV in the presence of methysergide. This increased PTV was also higher than the rise in the PTV evoked by caspofungin alone. (C) Recording of the PTV during exposure to the inhibitor of serotonin 5-HT_1A_ receptor WAY-100635 (20 μM). WAY-100635 had no effect on the PTV. Caspofungin effects on the PTV were not inhibited by WAY-100635. (♦, control; ▴, caspofungin; ●, WAY and caspofungin). (D) The application of WAY did not alter the PTV; however, caspofungin application following treatment with WAY-100635 induced a significant rise in the PTV. This rise was not different from that in the PTV seen under caspofungin application alone. (E) A selective inhibitor of 5-HT_2_, cyproheptadine, led to a prolonged increase in the PTV. Subsequent caspofungin application, still in the presence of cyproheptadine, induced a further increase in the PTV (●, control; ■, caspofungin; ▴, cyproheptadine and caspofungin). (F) Cyproheptadine induced a significant rise in the PTV, while the application of caspofungin in the presence of cyproheptadine induced a further significant rise in the PTV (caspo., caspofungin; cyproh., cyproheptadine; methys., methysergide; WAY, WAY-100635; horizontal bars in experimental recordings present exposure periods of defined pharmacological agents; *n*, number of individual experiments; ns, not significant; time scale bars presented in all individual panels; *, *P* < 0.05; **, *P* < 0.01; ***, *P* < 0.001; Mann-Whitney U test).

Since serotonin is involved in the modulation of murine tracheal mucociliary clearance via different serotonin receptors, we conducted a further series of experiments to prove the involvement of this signal pathway. Application of methysergide (100 μM), an unselective inhibitor of 5-hydroxytryptamine (5-HT) receptors 1 and 2 (5-HT_1,2_), did not alter the basal PTV (*P* = 0.93) (Fig. 3A and B). The application of caspofungin (60 μM) in the presence of methysergide evoked a significant rise in the PTV to 181% ± 9% (*n* = 7; *P* < 0.001) relative to methysergide alone (Fig. 3A and B). This rise in the PTV was also different from that observed under caspofungin application alone (*P* < 0.01); still, we observed a sustained rise in the PTV that remained at a plateau throughout the observation period.

WAY-100635 (50 μM), an inhibitor of 5-HT_1A_ receptors, also did not influence the PTV itself. When caspofungin was administered in the presence of WAY-100635, we observed a significant increase in the PTV to 160% ± 5.7% (*P* < 0.01 versus control) that was not different from that measured under caspofungin application alone (*P* = 0.135) (Fig. 3C and D).

In a further series of experiments, we inhibited 5-HT_2_ receptors prior to caspofungin application using the inhibitor cyproheptadine (2.5 μg/ml). Application of caspofungin in the presence of cyproheptadine induced a significant sustained increase in the PTV that was significantly higher than the increase in the PTV observed under cyproheptadine administration alone. This rise in the PTV was not different from the rise in the PTV that was evoked by caspofungin alone (Fig. 3E and F).

We found no evidence that caspofungin influenced the PTV via known membrane-bound receptors. Subsequently, we investigated whether caspofungin was able to induce the PTV by interfering with known intracellular signal cascades.

**FIG 4:**
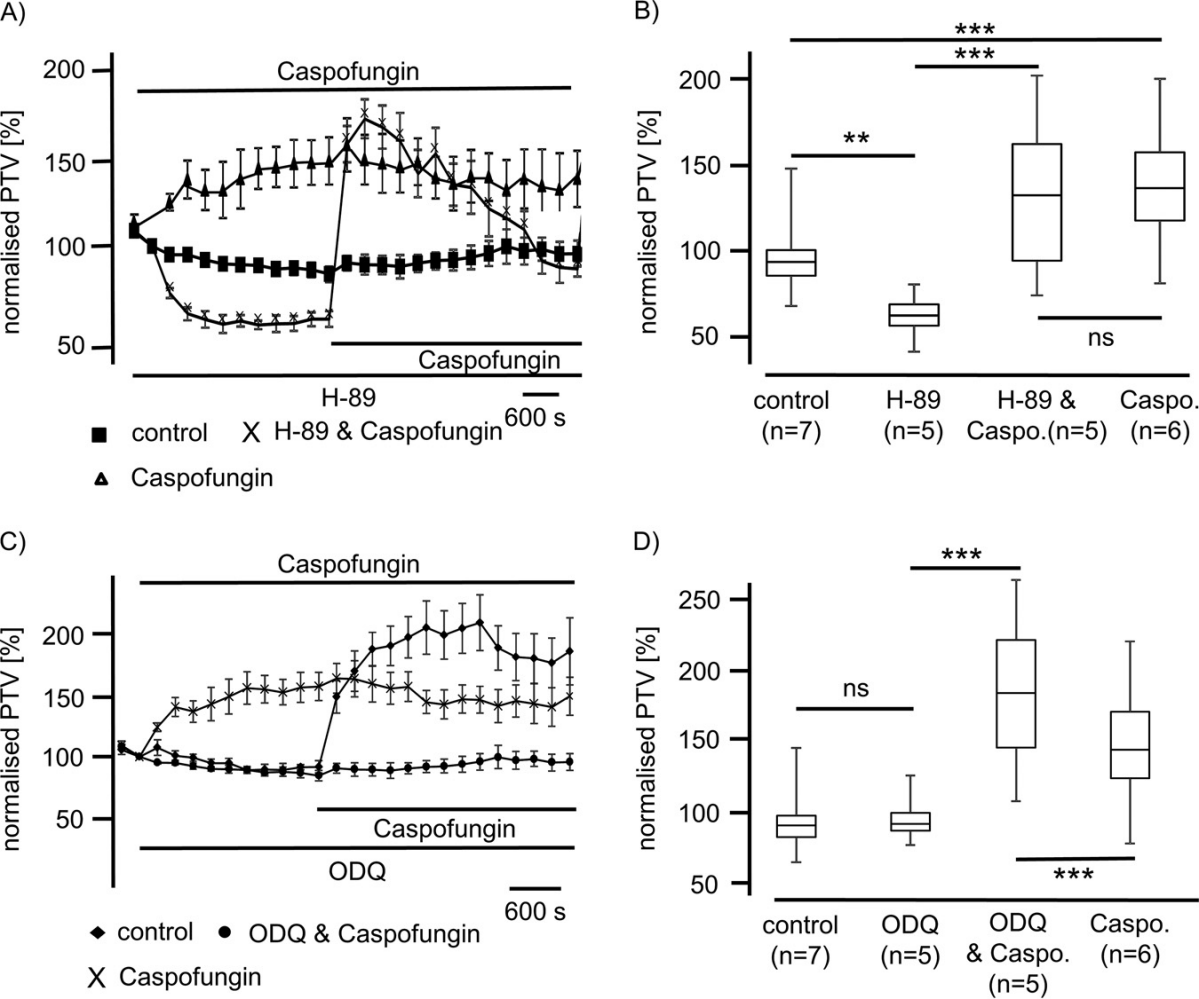
Effect of caspofungin is independent of protein kinase A (PKA) and guanylate cyclase reaction cascade. (A) The inhibition of PKA by application of H-89 (10 μM) significantly reduced the basal PTV. Administration of caspofungin in the presence of H-89 was immediately followed by a significant increase in the PTV. (B) The caspofungin-induced rapid increase in the PTV in the presence of H-89 was not different from the increase in the PTV evoked by caspofungin alone. (C) Inhibition of sGC using ODQ (10 μM) did not alter the PTV. Still, in the presence of ODQ, caspofungin application responded with a rapid and sustained increase in the PTV. (D) ODQ did not alter the PTV relative to control conditions. The response of the PTV to caspofungin in the presence of ODQ was higher than the response to caspofungin alone (caspo., caspofungin; horizontal bars in experimental recordings present exposure periods of defined pharmacological agents; *n*, number of individual experiments; ns, not significant; time scale bars are present in all individual panels; **, *P* < 0.01; ***, *P* < 0.001; Mann-Whitney U test).

Inhibition of the cyclic adenosine 3′,5′-monophosphate (cAMP)-dependent protein kinase signal pathway was achieved by the application of H-89 (10 μM) and reduced the baseline PTV to 59% (*n* = 5; *P* < 0.01) relative to control therapy. Meanwhile, caspofungin applied in the presence of H-89 significantly enhanced the PTV to a maximum of 168% ± 25% (*n* = 5; *P* < 0.001). This increase in the PTV evoked by caspofungin while inhibiting the cyclic AMP (cAMP)-dependent protein kinase signal pathway by H-89 was not different from the increase in the PTV triggered by caspofungin alone (154% ± 32%) (*n* = 6; *P* = 0.86) (Fig. 4A and B). However, it showed a slow decrease in the PTV at the end of the observation period.

We further examined whether caspofungin acts via a nitric oxide (NO) signal pathway to enhance the PTV. The possible interference of this signal pathway was verified by the inhibition of soluble guanylate cyclase (sGC) using the selective inhibitor 1H-[1,2,4]oxadiazolo[4,3-a]quinoxalin-1-one (ODQ) (10 μM). ODQ alone did not alter the basal PTV (89% ± 2.8%) (*n* = 5) after a 15-min incubation period relative to control therapy with resting for 15 min (87% ± 3.6%) (*n* = 7; *P* = 0.093). Caspofungin in the presence of ODQ induced a sustained rise in the PTV to 187% ± 15% (*n* = 5) that was significantly higher than the rise in the PTV induced by caspofungin alone (146% ± 8%) (*n* = 7; *P* < 0.001) (Fig. 4C and D). Thus, we found no evidence that caspofungin enhances the PTV via an sGC pathway in these cilia-bearing cells.

### Interference of ROS generation by caspofungin.

**FIG 5:**
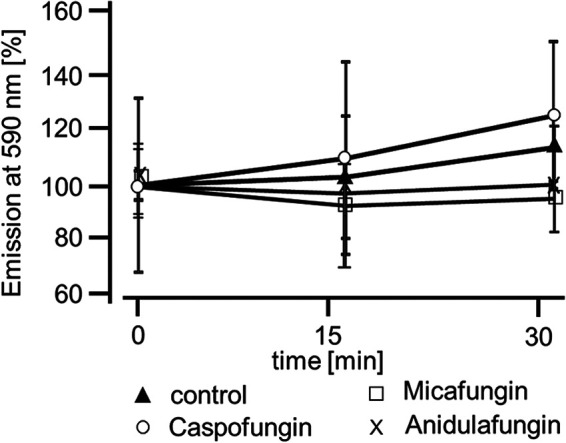
ROS generation in tracheal epithelial cells during the application of echinocandins. ROS were detected using the Amplex Red reagent for whole mouse tracheae. Fluorescence was measured at 590 nm for 30 min and expressed as a percentage of the initial fluorescence value. In untreated organs, only a slight change in the fluorescence intensity was recorded, suggesting a slight change in ROS generation. In tracheae exposed to either caspofungin, anidulafungin, or micafungin, we recorded only minute changes in ROS generation. Each organ was assessed at three consecutive time points, and statistical testing compared fluorescence recordings taken at 15 min and 30 min with the initial fluorescence (control, *n* = 4; caspofungin, *n* = 5; anidulafungin, *n* = 4; micafungin, *n* = 4; ●, control; ♦, caspofungin; □, anidulafungin; X, micafungin; Wilcoxon rank-sum test).

Since ROS generation changes cilia function and, here, interferes with signal pathway cascades, we elucidated whether caspofungin changes ROS generation in isolated mouse tracheae. We measured ROS generation using the aforementioned Amplex red assay kit. Fluorescence was recorded for 30 min under each condition, i.e., control therapy or echinocandin exposure. Under control conditions, the fluorescence showed only a trend of increase after an exposure period of 30 min to 126% ± 27% relative to the initial value (*n* = 4; *P* = 0.38). Tracheae exposed to caspofungin for 30 min also showed no significant alterations in ROS generation, 114% ± 12% (*n* = 5; *P* = 0.13), compared with the initial fluorescence value. The fluorescence under the application of anidulafungin or micafungin did not trigger ROS generation (anidulafungin 100.5% ± 3% [*n* = 4; *P* = 0.56]; micafungin 98.4% ± 1% [*n* = 4; *P *= 0.12]). There was no difference in ROS generation between control and echinocandin-treated organs at the end of the exposure period (caspofungin, *P* = 0.29; anidulafungin, *P* = 0.34; micafungin, *P* = 0.17) (Fig. 5).

### Ca^2+^-dependent effects of echinocandins on PTV. (i) Caspofungin.

**FIG 6:**
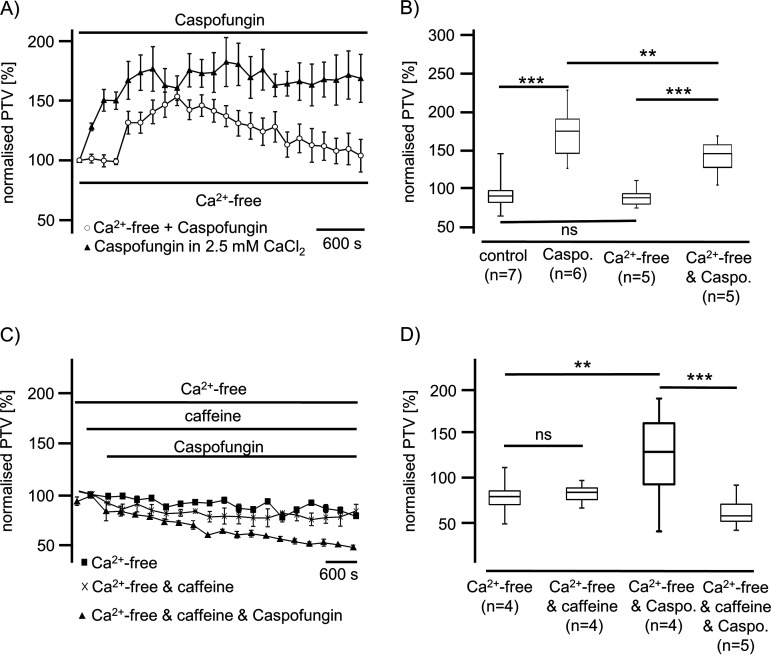
Caspofungin effects depend upon Ca^2+^ derived from caffeine-sensitive stores. (A) In Ca^2+^-free buffer medium, caspofungin induced a significant rise in the PTV that slowly decelerated during the exposure period. (B) The basal PTV was not affected by Ca^2+^-free buffer solutions (Wilcoxon rank-sum test). The significant rise in the PTV evoked by caspofungin in Ca^2+^-free buffer solution was significantly lower than that in Ca^2+^-containing buffer solution (Mann-Whitney U test). (C) Mouse tracheae exposed to caffeine (30 mM) in Ca^2+^-free buffer solution did not demonstrate alterations in the PTV. The application of caspofungin to tracheae primarily exposed to caffeine in Ca^2+^-free solution also did not evoke an increase in the PTV. Instead, the PTV slowly decelerated below the basal PTV value. (D) Caffeine (30 mM) in Ca^2+^-free medium did not change the PTV relative to the control in Ca^2+^-free solution (Mann-Whitney U test). The application of caspofungin in the presence of caffeine (30 mM) did not affect the basal PTV. In Ca^2+^-free buffer medium, the PTV level during caspofungin exposure in the presence of caffeine was significantly lower than that measured during sole caspofungin exposure. (■, Ca^2+^-free control buffer medium; ♦, Ca^2+^-free buffer medium with caspofungin; X, Ca^2+^-free buffer medium with caffeine; ▴, Ca^2+^-free buffer medium with caffeine and caspofungin; horizontal bars in experimental recordings present exposure periods of defined pharmacological agents; *n*, number of individual experiments; ns, not significant; time scale bars are present in all individual panels; **, *P* < 0.01; ***, *P* < 0.001; Mann-Whitney U test).

We further investigated whether the effects of echinocandins depend upon the cytosolic Ca^2+^ concentration ([Ca^2+^]_i_). Tracheae resting in Ca^2+^-free conditions showed no changes in the basal PTV, remaining constant during the entire observation period. However, the application of echinocandins in Ca^2+^-free buffer immediately triggered a significant rise in the PTV, reaching a peak of 153% ± 7% (*n* = 5; *P* < 0.001 versus control) (Fig. 6A and B), although the response to caspofungin in Ca^2+^-free buffer was significantly lower than that in Ca^2+^-containing buffer solution (182% ± 20%) (*n* = 6; *P* < 0.01).

The depletion of internal endoplasmic reticulum (ER) Ca^2+^ stores prior to caspofungin application was achieved by caffeine (30 mM). Caffeine exposure alone did not change the PTV (78% ± 1.2%) relative to Ca^2+^-free controls (82% ± 13%) (*P* = 0.23) (Fig. 6C). Exposure to caspofungin (60 μM) after the depletion of caffeine-sensitive Ca^2+^ stores did not increase the PTV (65% ± 13%), which was significantly lower than the PTV evoked by caspofungin alone in Ca^2+^-free solution (164% ± 20%) (*P* < 0.001) (Fig. 6C and D). Hence, the depletion of caffeine-sensitive Ca^2+^ stores completely inhibited the caspofungin effect on the PTV.

### (ii) Anidulafungin.

**FIG 7:**
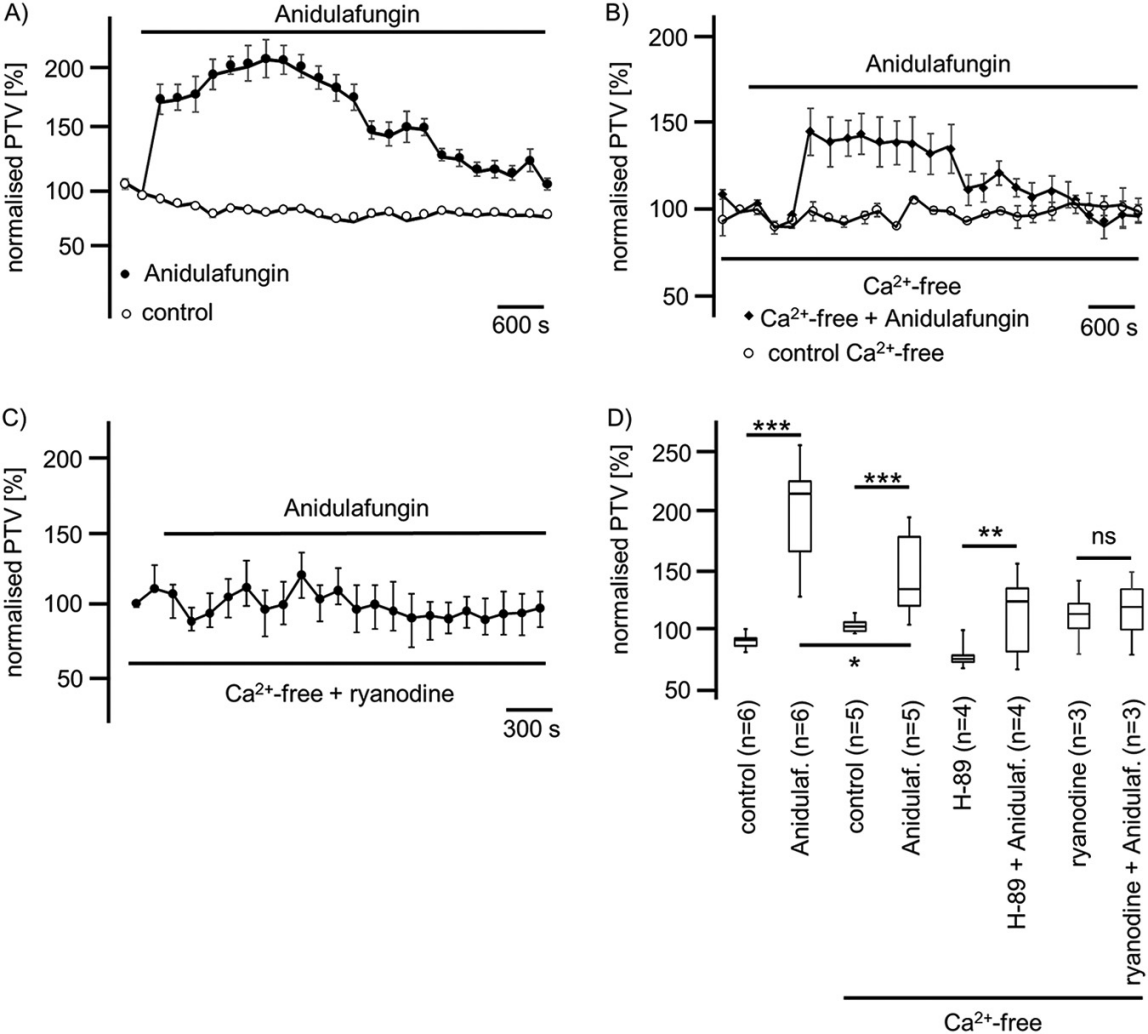
Anidulafungin induced the PTV in a manner dependent on external and internal Ca^2+^ concentrations. (A) In Ca^2+^-containing buffer solution, anidulafungin induced a prolonged rise in the PTV that reached more than 200% of the basal PTV. Under long-term exposure, we observed a decline in the PTV. (B) In Ca^2+^-free buffer solution, the anidulafungin-induced rise in the PTV was reduced to approximately 150% of the basal PTV. Still, this rise in the PTV was prolonged and showed a slow decay during the exposure period. (C) Inhibition of ryanodine receptors using ryanodine in a molecular range (40 μM) reduced anidulafungin-activated PTV. (D) Anidulafungin induced a significant increase of the PTV in Ca^2+^-containing or Ca^2+^-free buffer solutions; however, the increase in the PTV was significantly lower in the Ca^2+^-free solution. The inhibition of the PKA signal transduction pathway using H-89 did not prevent an anidulafungin-induced rise in the PTV, but ryanodine prevented the anidulafungin-induced rise in the PTV (horizontal bars in experimental recordings present exposure periods of defined pharmacological agents; *n*, number of individual experiments; ns, not significant; time scale bars are present in all individual panels; *, *P* < 0.05; **, *P* < 0.01; ***, *P* < 0.001; Mann-Whitney U test).

The application of anidulafungin (44 μM) in Ca^2+^-free buffer solution still evoked a significant rise in the PVT to 140% ± 16% (*n* = 5; *P* < 0.001); however, this accelerated PTV was lower than that in Ca^2+^-containing buffer solution (210% ± 19%) (*n* = 6; *P* < 0.05) (Fig. 7). Still, during the inhibition of the PKA signal pathway by H-89 (10 μM), the application of anidulafungin (44 μM) evoked a significant rise in the PTV to 136% ± 5% (*n* = 4; *P* < 0.01) (Fig. 7D). A high concentration of ryanodine (40 μM) was used to inhibit ryanodine receptors of the ER. Subsequent prolonged exposure to anidulafungin (44 μM) evoked no change in the PTV (119% ± 8%) (*n* = 3; *P* = 0.34) compared with ryanodine (116% ± 7%) (*n* = 3) (Fig. 7C and D).

### (iii) Micafungin.

**FIG 8:**
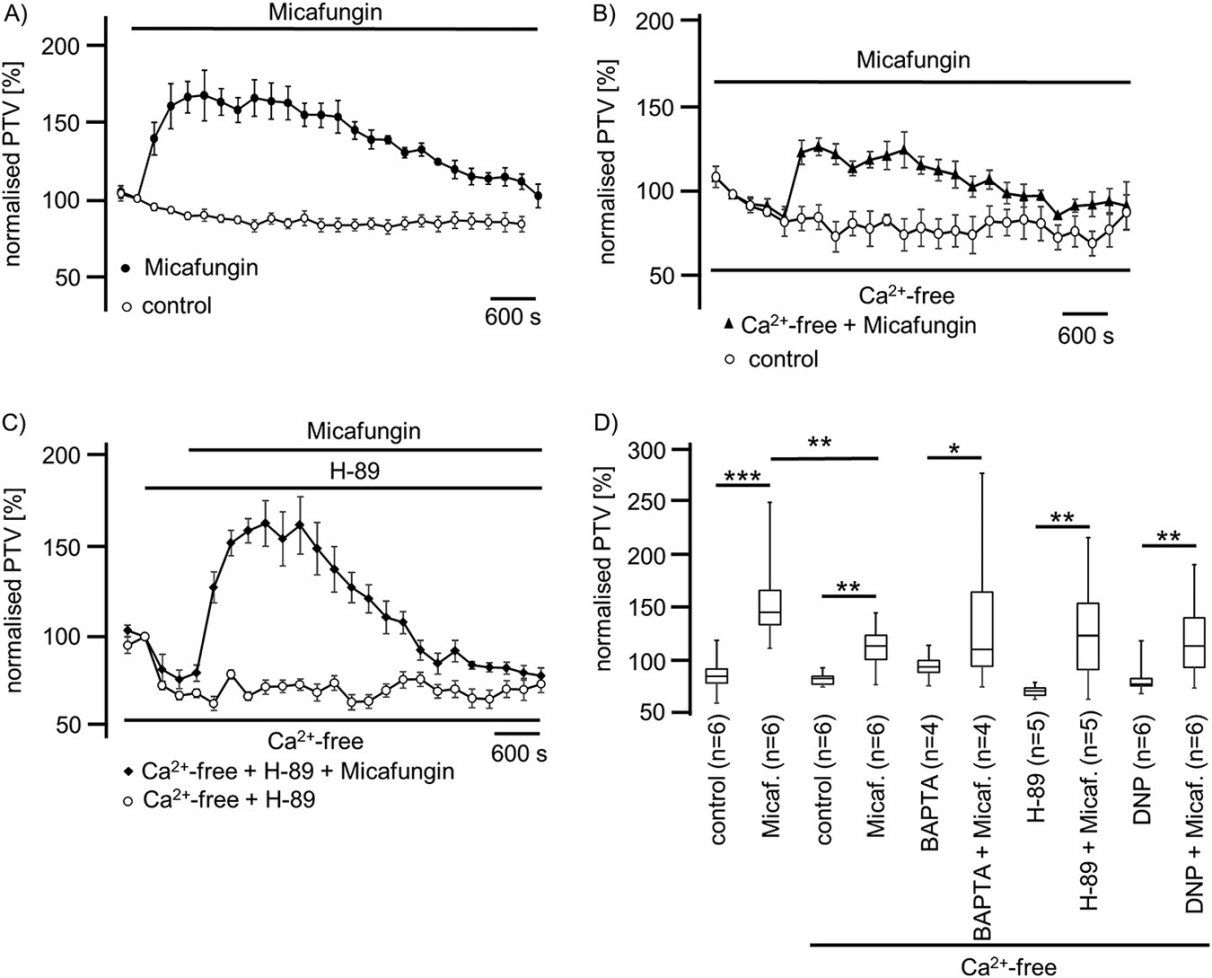
Micafungin evokes a prolonged rise in the PTV partly dependent on extracellular Ca^2+^. (A) In Ca^2+^-containing solution, micafungin induced a prolonged rise in the PTV, up to 165% of the basal PTV. The PTV slowly declined under long-term micafungin exposure. (B) The micafungin-induced rise in the PTV was partly dependent on the extracellular Ca^2+^ concentration, since the observed rise in the PTV was reduced in Ca^2+^-free buffer solution. Still, under prolonged micafungin exposure, the PTV declined. (C) The inhibition of PKA using H-89 reduced the basal PTV, while subsequent application of micafungin induced a significant rise in the PTV, which declined after reaching a maximum velocity. (D) Micafungin induced a significant rise in the PTV either in Ca^2+^-containing or Ca^2+^-free medium. However, the PTV in Ca^2+^-free buffer solution was significantly lower than that in Ca^2+^-containing buffer solution. In tracheae treated with BAPTA-AM to reduce [Ca^2+^]_i_, micafungin induced a significant transient rise in the PTV. H-89, the inhibitor of the PKA signal transduction pathway, did not prevent the micafungin-induced rise in the PTV. The depletion of mitochondrial Ca^2+^ stores and simultaneous interruption of mitochondrial ATP synthesis using DNP did not prevent the micafungin-evoked rise in the PTV (horizontal bars in experimental recordings present exposure periods of defined pharmacological agents; *n*, number of individual experiments; time scale bars are present in all individual panels; *, *P* < 0.05; **, *P* < 0.01; ***, *P* < 0.001; Mann-Whitney U test).

Micafungin (50 μM) induced a rise in the PTV up to 165% ± 16% (*n* = 6) in Ca^2+^-containing solution, which was reduced in Ca^2+^-free solution to 126% ± 10% (*n* = 6; *P* < 0.01) (Fig. 8A, B, and D). The evoked PTV slowly decelerated during the exposure period (Fig. 8A). In tracheae treated with BAPTA-AM [1,2-bis(2-aminophenoxy)ethane-*N*,*N*,*N′*,*N*′-tetraacetic acid tetrakis(acetoxymethyl ester)], the application of micafungin (50 μM) still evoked a transient rise in the PTV to a maximum of 145% ± 24% (*n* = 4; *P* < 0.05) (Fig. 8D). The inhibition of the PKA signal pathway by H-89 did not prevent a micafungin-induced rise in the PTV of 162% ± 16% (*n* = 5; **, *P* < 0.01) (Fig. 8C and D). The depletion of mitochondrial Ca^2+^ stores by the application of DNP (125 μM), an uncoupler of the mitochondrial respiratory chain, reduced the PTV to 74% ± 2% (*n* = 6). The subsequent application of micafungin (50 μM) significantly enhanced the PTV to 133% ± 25% (*n* = 6; *P* < 0.01) (Fig. 8D) relative to DNP alone.

**FIG 9:**
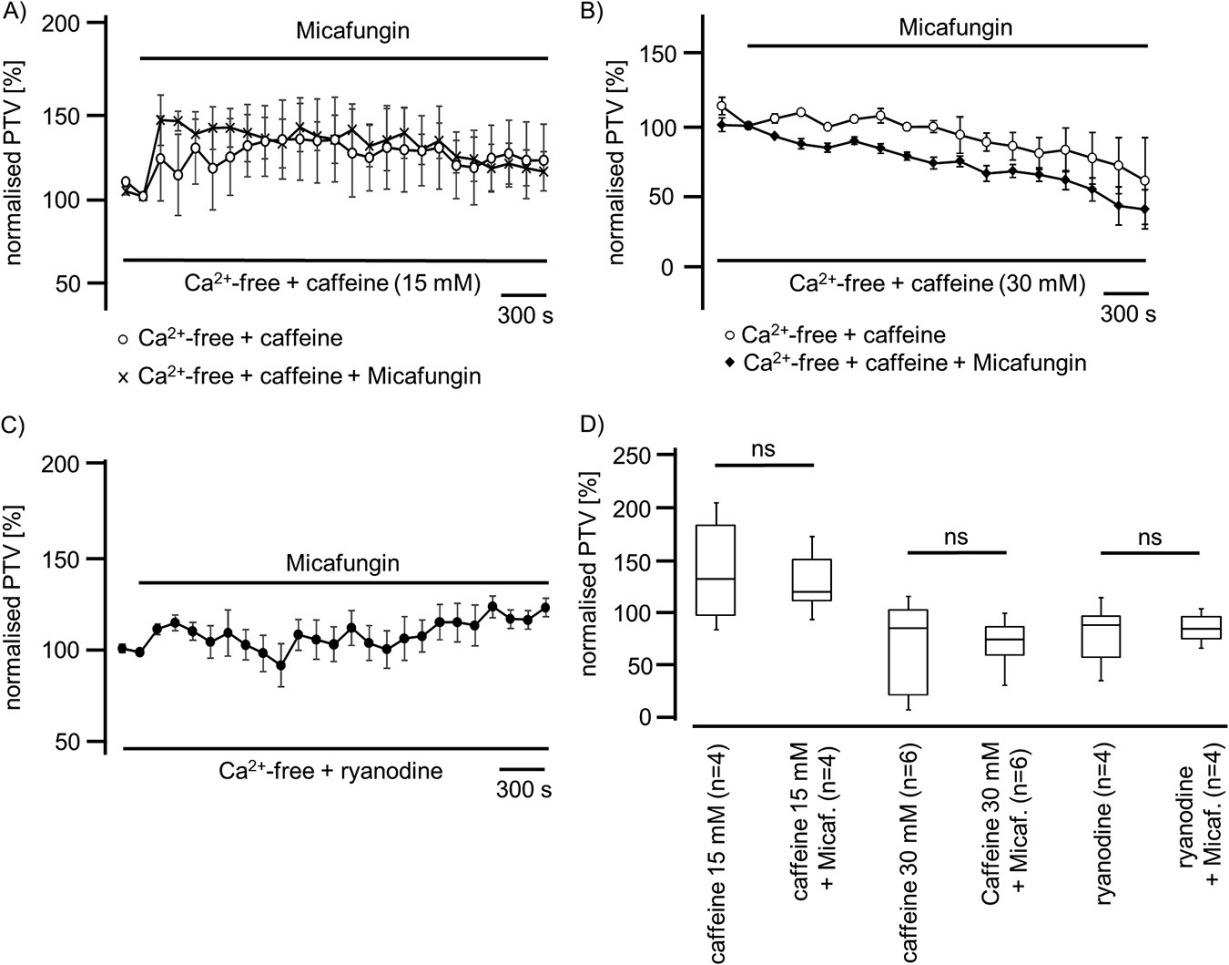
Micafungin induced the PTV in a manner dependent on ER Ca^2+^ stores. (A) The exposure of mouse tracheae to low-dose caffeine (15 mM) induced a sustained rise in the PTV that was not further increased by micafungin application. (B) A high caffeine concentration (30 mM) did not trigger a PTV increase, and subsequent application of micafungin also did not provoke a rise in the PTV. (C) Ryanodine at a high concentration (40 μM) prevented the micafungin-induced rise in the PTV. (D) A low caffeine concentration (15 mM) evoked a rise in the PTV that was not further enhanced in the presence of micafungin. A high caffeine concentration (30 mM) did not accelerate the PTV, and subsequent application of micafungin did not change the PTV. A high ryanodine concentration did not evoke an increase in the PTV, and successive micafungin application did not increase the PTV (horizontal bars in experimental recordings present exposure periods of defined pharmacological agents; *n*, number of individual experiments; ns, not significant; time scale bars in all individual panels; Mann-Whitney U test).

Further, we investigated whether Ca^2+^ stores in the ER contribute to micafungin-induced PTV. We depleted ER Ca^2+^ stores using caffeine. Under low-dose caffeine (15 mM) application, we then measured an increase in the PTV that was not further enhanced under micafungin exposure (Fig. 9A). A higher caffeine concentration (30 mM) did not induce the PTV, which also was not induced by subsequent micafungin application (Fig. 9B). The inhibition of the ryanodine receptors by ryanodine (120 μM) almost completely prevented the PTV response to micafungin (Fig. 9C and D).

## DISCUSSION

In the present study, we gained evidence that echinocandins increase the PTV in intact murine tracheal epithelia. Furthermore, we investigated the possible signal cascades of echinocandin-induced alteration of mice tracheal epithelial function and revealed that they alter the PTV via intracellular Ca^2+^-dependent signal cascades in a dose-dependent manner. The mucociliary clearing system is an important mechanism that functions to disburden the airway surface area of the lung from debris and pathogen particles (49, 50). Besides bacterial and viral pathogens as well as the fungal cell wall, the polysaccharides galactomannan and zymosan can inhibit the PTV via reversible ROS generation in the tracheal tissue (51). Echinocandins are antifungal agents recommended for the empirical and specific treatment of invasive *Candida* and *Aspergillosis* infections and are widely used in clinical practice (52, 53).

First of all, we analyzed the impact of the three existing echinocandins (caspofungin, anidulafungin, and micafungin) on the PTV in murine tracheal epithelia and found that application of caspofungin, anidulafungin, or micafungin significantly increased the PTV (Fig. 1A). The effects of all three echinocandins appeared to be similar in a dose-dependent manner. Thus, we elucidated according to the signal pathway how echinocandins enhance the PTV; specifically, different signal pathways are described to alter the mouse tracheal PTV, including membrane-bound receptors, intracellular signal cascades, ROS generation, and intracellular Ca^2+^ homeostasis (13, 51). Subsequently, we used pharmacological inhibitors to identify the underlying mechanism of a caspofungin-induced increase of the PTV and whether membrane-bound receptors are involved in echinocandin-induced PTV. Since all three echinocandins triggered a similar response in the PTV when known plasma membrane-bound receptors are pharmacologically inhibited, here, we only present data for caspofungin. First, the potential effect on muscarinic receptors was investigated via the application of atropine, prior to caspofungin application, but our data revealed that caspofungin did not alter the PTV via muscarinic receptor activation. Second, the inhibition of beta-receptors did not alter the caspofungin-induced increase in the PTV. Third, neither the inhibition of 5-HT1 nor the inhibition of 5-HT2 receptors affected the rise of the PTV evoked by caspofungin.

Accordingly, we assumed that caspofungin did not influence the PTV via known membrane-bound receptors and subsequently investigated whether caspofungin could induce the PTV by interfering with intracellular signal cascades. Further inhibition of the cAMP-dependent protein kinase signal pathway, which is a cornerstone of signal transduction in tracheal epithelial cells, did not affect echinocandin-evoked activation in the PTV.

We further investigated whether echinocandins act via a NO signal pathway to enhance the PTV. It is known that NO spontaneously generated by airway epithelial cells plays a modulatory role in beta-adrenoreceptor–mediated stimulation of ciliary motility (54, 55). In the present study, we found no evidence that endogenously generated NO contributed to echinocandin-induced acceleration of the PTV. To elucidate this, we pharmacologically inhibited the NO signal transduction pathway prior to echinocandin application. This was achieved by the inhibition of sGC, the known target molecule of NO in the tracheal epithelium (56, 57), although we observed no alteration in the PTV under the subsequent echinocandin exposure. Although intrinsic generated NO plays a key role in the modulation of beta-adrenergic stimulation of ciliary motility, the observed echinocandin effect was not influenced or amplified by NO.

Since ROS generation impairs airway cilia function, we furthermore investigated whether echinocandins alter ROS generation in isolated mouse tracheae to accomplish an enhanced ciliary motility (18, 58). In experiments using whole mouse tracheae, we found no alterations in ROS generation during echinocandin exposure. We revealed that there was no difference in ROS generation at the end of the exposure period between control and echinocandin-treated organs. Consequently, we assume that echinocandins do not act via the inhibition of an ROS-sensitive pathway to provoke the PTV acceleration. Many of the established signal transduction cascades in ciliary cells investigated here depend upon an increased [Ca^2+^]_i_ (15, 30, 59, 60). Therefore, we investigated whether the echinocandin effect also depends upon homeostasis of [Ca^2+^]_i_. However, the increase in the PTV was only partly dependent on extracellular calcium. We assume that Ca^2+^ channels of the plasma membrane were activated directly or indirectly by echinocandins. Store-operated Ca^2+^ entry (SOCE) is a common mechanism in many cells to regulate cell function (61). Calcium influx via SOCE is mediated by STIM-operated ORAI channels (62–64) and may also be involved in Ca^2+^ influx to activate the PTV and replenish Ca^2+^ stores. Another important role could concern the involvement of the transient receptor potential vanilloid 4 cation channel (TRPV4) (65–67), a Ca^2+^-permeable nonselective cation channel in airway epithelial cells, which can also interact with calmodulin or other calcium-sensing molecules (68). Without the presence of extracellular Ca^2+^, TRPV4 currents are diminished (69), which may support our PTV measurement findings. In Ca^2+^-free buffer, the echinocandin-initiated rise in the PTV was as rapid as that in calcium-containing buffer solutions; however, it was still significantly reduced, leading to the assumption of the participation of TRPV4. These kinetics were similar to those observed in previous investigations (30). The subsequent rundown of the PTV in Ca^2+^-free buffer, still in the presence of echinocandins, could also be caused by a slow efflux of Ca^2+^ ions from the cytosol, and the reduced [Ca^2+^]_i_ may lead to an inactivation of the signal transduction cascade. The response to caspofungin and micafungin was completely prevented when ER Ca^2+^ stores were depleted by caffeine prior to their application. In experiments using ryanodine to inhibit ER-bound ryanodine receptors, the response in the PTV to micafungin or anidulafungin was prevented. Therefore, we assume that echinocandins release Ca^2+^ ions from the ER, which further activates signal transduction cascades to increase the ciliary beat frequency, propelling the PTV. We gained no evidence of the contribution of mitochondrial Ca^2+^ stores. Still, while we cannot say whether the increase in the PTV evoked by echinocandins is the consequence of direct liberation of Ca^2+^ ions from the ER and activation of store-operated channels of the plasma membrane, we can conclude that the activation mechanism is located downstream of the cAMP-dependent PKA (Fig. 4A, 7, and 8). The PKA is involved in the liberation of Ca^2+^ ions from the ER (30). However, in a study on isolated human tracheal epithelial cells, we previously demonstrated that caspofungin liberates Ca^2+^ from ER stores via ryanodine receptors (70). To elucidate further the underlying mechanisms, we will focus on store-operated Ca^2+^ channels, assessing how echinocandins activate ryanodine receptors or how they are involved in alternative Ca^2+^ liberation from the ER. Additionally, it has to be investigated if the echinocandin-induced PTV alteration also occurs in human tracheal epithelium. We know that the cilia function in mammals is similar, but it is difficult to establish an equivalent human model to examine the mucociliary clearance in intact human tracheal epithelium. To approach clinical *in vivo* conditions, an initial way is to examine cultured human ciliated tracheal epithelial cells that were harvested during bronchoalveolar lavage. However, this model discounts cell-to-cell signaling and the coordinated ciliary beat function of adjacent epithelial cells. Thus, further clinical studies are necessary to investigate whether patients receiving echinocandins for antifungal therapy reveal pulmonary benefits like lower rates of or faster recovery from pulmonary infections.

### Conclusions.

The echinocandins caspofungin, anidulafungin, and micafungin provoked a sustained elevation in the PTV that strongly depended upon extracellular Ca^2+^ and the content of internal caffeine- and ryanodine-sensitive ER stores. We can rule out any echinocandin interference with membrane-bound receptors or with further downstream-located signal transduction cascades. In mouse tracheae, echinocandins did not alter ROS generation, which is assumed to inhibit ciliary function. The mechanism of how echinocandins increase the PTV via Ca^2+^ influx or liberation ought to be further investigated to understand its interactions with mammalian cell organelles. Finally, experimental investigations on human tracheal epithelium and clinical studies are needed to clarify if these findings in echinocandin administration lead to a noticeable benefit for patients in the ICU.

## MATERIALS AND METHODS

### Mouse tracheal segment preparation and imaging.

For the present study, we used male C57BL6J mice aged 12 to 15 weeks (weighing 25 to 35 g) delivered by Charles Rivers (Sulzfeld, Germany). All experiments were performed according to the German guidelines for the care and use of laboratory animals. The study protocol was approved by the local committee for animal care of Justus Liebig University Giessen (Giessen, Germany) (permit no. 443_M 932). Study animals were sacrificed by inhalation of an overdose of isoflurane (Baxter, Unterschleissheim, Germany) in a closed chamber. All of the following preparation steps were performed within 30 min after euthanasia. Immediately after confirmation of death, we removed the trachea with a parasternal incision of the thorax and a median incision of the throat. Still *in situ*, the trachea was gently disconnected by slicing cranial to its bifurcation and directly caudal to the larynx. Immediately afterward, the organ was transferred into a Delta T culture dish (Bioptechs, Butler, PA, USA) containing 2 ml of preheated (30°C) 4-(2-hydroxyethyl)-1-piperazine ethanesulfonic acid (HEPES) buffer (pH 7.4). The bottom of the dish had previously been coated with Sylgard polymer (Dow Corning, Wiesbaden, Germany) to attain precise positioning of the trachea. Here, we fixed the submerged organs with two fine minutiae (Fiebig Lehrmittel, Berlin, Germany) so that the cartilage arches faced the Sylgard polymer and the musculus trachealis faced upwards; this position enabled the following fine preparation. Connective tissue and surrounding blood vessels were gently removed by employing spring scissors (Vannas-Tübingen; FST, Heidelberg, Germany). Finally, the pars membranacea, including the musculus trachealis, was cut open in the longitudinal direction, allowing for direct visualization of the respiratory epithelium. HEPES buffer was then replaced by 2 ml of fresh HEPES buffer (pH 7.4; 30°C). The dish containing the trachea was transferred to the stage-holder of an upright transmission light microscope (BX50 WI; Olympus, Hamburg, Germany). The stage-holder of the microscope was equipped with a temperature control unit that allowed the operator to maintain a constant temperature of 30°C in the center of the buffer solution throughout each experiment. In our experiments the temperature was set to 30°C after measuring the CBF and PTV during different temperatures, reaching from 22 to 42°C (data not published). Here, the best measuring conditions and imaging, depending on the camera resolution, could be achieved at 30°C. Therefore, we have to note that the measured PTV in our experiments might be slightly slower than real time, while the PTV is temperature dependent and accelerates with increasing temperatures. Imaging was performed using the TiLLvisION imaging software program (Till Photonics, Gräfeling, Germany).

### Measurement of PTV.

For the measurement of the PTV, we used 4 μl of Dynabeads (mean diameter, 2.8 to 4.5 μm; Dynal Biotech GmbH, Hamburg, Germany) added prior to each experiment into the bath solution. Dynabeads directly floating on the tracheal epithelium were focused in a bright-field mode between two cartilages using a 20× water immersion lens (BW50 WI; Olympus, Hamburg, Germany). At certain time points, we recorded short movie sequences with high sampling rates. Subsequently, the movie sequences were analyzed using the Image Pro Plus software (Media Cybernetics, Rockville, MD, USA). The viability of the tracheal ciliated cells was confirmed at the end of each experiment by the application of ATP (100 μM), leading to a maximal increase in the PTV. Only experiments responding to ATP with an increase in the PTV were included for further data processing. For each individual experiment, we evaluated approximately 200 to 400 particle tracks at certain predefined time points. At these time points, we acquired 200 images during a period of 16.726 s. Further, nonmoving objects were excluded and background subtraction was performed pixel by pixel (200 images/16.726 s = 1 image/83.63 ms). Following the background subtraction, the images were converted and the formerly dark polystyrene Dynabeads now appeared as bright images. Only particles that displayed a lateral deviation of less than 15% were selected for further data acquisition and evaluation. These images were transformed into a binary system with a threshold allowing for the discrimination between bright bead images and the dark background. Finally, on a greyscale, we reduced the 12-bit film to an eight-bit film so that we could monitor individual particles using the TiLLvisION software (Till Photonics, Gräfeling, Germany). From these measurements, we calculated an average PTV.

### Estimation of ROS.

To gain evidence for ROS generation from the trachea, we employed the Amplex red hydrogen peroxide/peroxidase assay kit (Life Technologies, Carlsbad, CA, USA) for the detection of H_2_O_2_. Freshly harvested tracheae were prepared as described above and equilibrated in fresh HEPES buffer (2 ml, 30°C, pH 7.4) for 60 min. After this equilibration period, we added 20 μl of H_2_O for control experiments (*n* = 4) or 120 μM caspofungin, 44 μM anidulafungin, 50 μM micafungin (each diluted in 20 μl of H_2_O, *n* = 5) to the bath solution. The fluorescence sampling was started directly following the administration of echinocandins (60 min after tissue preparation), after 75 min (15 min after echinocandin administration), and after 90 min (30 min after echinocandin administration). The tracheal tissue specimens were incubated with 10 μM Amplex red reagent (10-acetyl-3,7-dihydroxyphenoxazine) to detect released H_2_O_2_. The emitted fluorescence was measured with a microplate reader equipped for excitation wavelengths in the range of 530 to 560 nm and the detection of fluorescence emission wavelength at ∼590 nm (Infinite M200; Tecan, Maennedorf, Switzerland). For data processing, fluorescence was normalized and initial fluorescence was arbitrarily set as 100%.

### Drugs and buffer solutions.

The following drugs were administered: ATP (100 μM, Sigma-Aldrich, St. Louis, MO, USA), caspofungin (60 μM or 120 μM diluted in 20 μl of H_2_O; MSD, Kenilworth, NJ, USA), anidulafungin (44 μM diluted in 20 μl of H_2_O; Pfizer, New York, NY, USA), micafungin (50 μM diluted in 20 μl of H_2_O; Astellas Pharma GmbH, Munich, Germany), atropine (10 μM; B.Braun, Melsungen, Germany), ICI-118,551 (10 μM; Sigma-Aldrich, St. Louis, MO, USA), caffeine (30 mM; Roth, Karlsruhe, Germany), WAY-100635 (20 μM, diluted in 20 μl of H_2_O; Sigma-Aldrich, St. Louis, MO, USA), ryanodine (40 μM, diluted in 25 μl of H_2_O; Bio-Techne GmbH, Wiesbaden-Nordenstadt, Germany), H-89 (10 μM; Sigma-Aldrich, St. Louis, MO, USA), methysergide (100 μM; Sigma-Aldrich, St. Louis, MO, USA). Cyproheptadine (2.5 μg/ml; Sigma-Aldrich, St. Louis, MO, USA), 1H-[1,2, 4]oxadiazolo[4,3-a]quinoxalin-1-one (ODQ) (10 μM; Sigma-Aldrich, St. Louis, MO, USA), or 2,4-dinitrophenol (DNP) (125 μM; Sigma-Aldrich, St. Louis, MO, USA) was diluted in ethanol for stock solution. H-89, methysergide, and ODQ were diluted in dimethyl sulfoxide. End concentrations were achieved by applying the stock solution directly to the buffer solution in the recording chamber. Dimethyl sulfoxide did not exceed volumes of 20 μl in 2 ml of HEPES to prevent the induction of unspecific disturbances of cilia-bearing cells. Cyproheptadine was diluted in ethanol for the stock solution. All preparations and experiments were carried out in HEPES solution consisting of 20 mM HEPES, 4.5 mM KCl, 2.5 mM CaCl_2_, 11 mM glucose, 140 mM NaCl, and 1 mM MgCl_2_; the pH was adjusted using NaOH (4 M) to 7.4 at 30°C for tissue preparation. For Ca^2+^-free solutions, CaCl_2_ was replaced by 1 mM ethylene glycol tetraacetic acid. As a balance in buffer solutions with KCl concentrations of 30 mM, the NaCl concentration was reduced by the same amount. Solutions containing 30 mM KCl also contained 2.5 mM CaCl_2_.

### Statistical analysis.

Only tracheal preparations were included in further statistical evaluation that responded to ATP application at the end of each individual recording. The Mann-Whitney U test was used to compare equivalent measuring points from different experiments. The Wilcoxon rank-sum test was used to compare paired variables. EC_50_ (median effective concentration) values were calculated using the Hill equation. Statistical data evaluation and testing were performed using the GraphPad Prism software (version 5.04; GraphPad Software, La Jolla, CA, USA).

## References

[1] Stuart BO. 1984. Deposition and clearance of inhaled particles. Environ Health Perspect 55:369–390. doi:10.1289/ehp.8455369. 6376108 PMC1568355

[2] Guirao B, Joanny JF. 2007. Spontaneous creation of macroscopic flow and metachronal waves in an array of cilia. Biophys J 92:1900–1917. doi:10.1529/biophysj.106.084897. 17189311 PMC1861806

[3] Gheber L, Priel Z. 1989. Synchronization between beating cilia. Biophys J 55:183–191. doi:10.1016/S0006-3495(89)82790-0. 2930819 PMC1330453

[4] Rhodin JA. 1966. The ciliated cell. Ultrastructure and function of the human tracheal mucosa. Am Rev Respir Dis 93(Suppl):1–15. doi:10.1164/arrd.1966.93.3P2.1. 5954680

[5] Dalhamn T. 1956. Mucous flow and ciliary activity in the trachea of healthy rats and rats exposed to respiratory irritant gases (SO2, H3N, HCHO): a functional and morphologic (light microscopic and electron microscopic) study, with special reference to technique. Acta Physiol Scand Suppl 36:1–161. 13326492

[6] Sanderson MJ, Sleigh MA. 1981. Ciliary activity of cultured rabbit tracheal epithelium: beat pattern and metachrony. J Cell Sci 47:331–347. doi:10.1242/jcs.47.1.331. 7263784

[7] Fujiwara K, Hakansson CH, Toremalm NG. 1972. Studies on the physiology of the trachea. VI. Interaction between ciliary beat frequency and transport of secretions. Ann Otol Rhinol Laryngol 81:212–217. doi:10.1177/000348947208100206. 5027590

[8] Melville GN, Iravani J. 1975. Factors affecting ciliary beat frequency in the intrapulmonary airways of rats. Can J Physiol Pharmacol 53:1122–1128. doi:10.1139/y75-156. 1222380

[9] van de Donk HJ, Zuidema J, Merkus FW. 1980. The influence of the pH and osmotic pressure upon tracheal ciliary beat frequency as determined with a new photo-electric registration device. Rhinology 18:93–104. 7403788

[10] Wyatt TA. 2015. Cyclic GMP and cilia motility. Cells 4:315–330. doi:10.3390/cells4030315. 26264028 PMC4588039

[11] Davis SD, Rosenfeld M, Lee H-S, Ferkol TW, Sagel SD, Dell SD, Milla C, Pittman JE, Shapiro AJ, Sullivan KM, Nykamp KR, Krischer JP, Zariwala MA, Knowles MR, Leigh MW. 2019. Primary ciliary dyskinesia: longitudinal study of lung disease by ultrastructure defect and genotype. Am J Respir Crit Care Med 199:190–198. doi:10.1164/rccm.201803-0548OC. 30067075 PMC6353004

[12] Bustamante-Marin XM, Ostrowski LE. 2017. Cilia and mucociliary clearance. Cold Spring Harb Perspect Biol 9:a028241. doi:10.1101/cshperspect.a028241. 27864314 PMC5378048

[13] Weiterer S, Schulte D, Müller S, Kohlen T, Uhle F, Weigand MA, Henrich M. 2014. Tumor necrosis factor alpha induces a serotonin dependent early increase in ciliary beat frequency and epithelial transport velocity in murine tracheae. PLoS One 9:e91705. doi:10.1371/journal.pone.0091705. 24626175 PMC3953516

[14] Khan AR, Bengtsson B, Lindberg S. 1986. Influence of substance P on ciliary beat frequency in airway isolated preparations. Eur J Pharmacol 130:91–96. doi:10.1016/0014-2999(86)90186-X. 2430817

[15] Sanderson MJ, Dirksen ER. 1986. Mechanosensitivity of cultured ciliated cells from the mammalian respiratory tract: implications for the regulation of mucociliary transport. Proc Natl Acad Sci USA 83:7302–7306. doi:10.1073/pnas.83.19.7302. 3463968 PMC386704

[16] Kobayashi K, Tamaoki J, Sakai N, Kanemura T, Horii S, Takizawa T. 1990. Angiotensin II stimulates airway ciliary motility in rabbit cultured tracheal epithelium. Acta Physiol Scand 138:497–502. doi:10.1111/j.1748-1716.1990.tb08877.x. 2162128

[17] Perniss A, Liu S, Boonen B, Keshavarz M, Ruppert A-L, Timm T, Pfeil U, Soultanova A, Kusumakshi S, Delventhal L, Aydin Ö, Pyrski M, Deckmann K, Hain T, Schmidt N, Ewers C, Günther A, Lochnit G, Chubanov V, Gudermann T, Oberwinkler J, Klein J, Mikoshiba K, Leinders-Zufall T, Offermanns S, Schütz B, Boehm U, Zufall F, Bufe B, Kummer W. 2020. Chemosensory cell-derived acetylcholine drives tracheal mucociliary clearance in response to virulence-associated formyl peptides. Immunity 52:683–699. doi:10.1016/j.immuni.2020.03.005. 32294408

[18] Burman WJ, Martin WJ. 1986. Oxidant-mediated ciliary dysfunction. Possible role in airway disease. Chest 89:410–413. doi:10.1378/chest.89.3.410. 3948555

[19] Sutto Z, Conner GE, Salathe M. 2004. Regulation of human airway ciliary beat frequency by intracellular pH. J Physiol 560:519–532. doi:10.1113/jphysiol.2004.068171. 15308676 PMC1665258

[20] Kilgour E, Rankin N, Ryan S, Pack R. 2004. Mucociliary function deteriorates in the clinical range of inspired air temperature and humidity. Intensive Care Med 30:1491–1494. doi:10.1007/s00134-004-2235-3. 15024566

[21] Nikolaizik W, Hahn J, Bauck M, Weber S. 2020. Comparison of ciliary beat frequencies at different temperatures in young adults. ERJ Open Res 6:00477-2020. doi:10.1183/23120541.00477-2020. 33263055 PMC7682707

[22] Simet SM, Pavlik JA, Sisson JH. 2013. Dietary antioxidants prevent alcohol-induced ciliary dysfunction. Alcohol 47:629–635. doi:10.1016/j.alcohol.2013.09.004. 24169090 PMC3850762

[23] Jiao J, Wang H, Meng N, Zhang L. 2012. Different cilia response to adenosine triphosphate or benzalkonium chloride treatment in mouse nasal and tracheal culture. J Otorhinolaryngol Relat Spec 74:280–285. doi:10.1159/000343800. 23154526

[24] König P, Krain B, Krasteva G, Kummer W. 2009. Serotonin increases cilia-driven particle transport via an acetylcholine-independent pathway in the mouse trachea. PLoS One 4:e4938. doi:10.1371/journal.pone.0004938. 19290057 PMC2654158

[25] Zhang L, Han D, Sanderson MJ. 2005. Effect of isoproterenol on the regulation of rabbit airway ciliary beat frequency measured with high-speed digital and fluorescence microscopy. Ann Otol Rhinol Laryngol 114:399–403. doi:10.1177/000348940511400512. 15966529

[26] Matsuura S, Shirakami G, Iida H, Tanimoto K, Fukuda K. 2006. The effect of sevoflurane on ciliary motility in rat cultured tracheal epithelial cells: a comparison with isoflurane and halothane. Anesth Analg 102:1703–1708. doi:10.1213/01.ane.0000216001.36932.a3. 16717313

[27] Frohock JI, Wijkstrom-Frei C, Salathe M. 2002. Effects of albuterol enantiomers on ciliary beat frequency in ovine tracheal epithelial cells. J Appl Physiol 92:2396–2402. doi:10.1152/japplphysiol.00755.2001. 12015353

[28] Lieb T, Frei CW, Frohock JI, Bookman RJ, Salathe M. 2002. Prolonged increase in ciliary beat frequency after short-term purinergic stimulation in human airway epithelial cells. J Physiol 538:633–646. doi:10.1113/jphysiol.2001.013222. 11790825 PMC2290065

[29] Taira M, Tamaoki J, Nishimura K, Nakata J, Kondo M, Takemura H, Nagai A. 2002. Adenosine A(3) receptor-mediated potentiation of mucociliary transport and epithelial ciliary motility. Am J Physiol Lung Cell Mol Physiol 282:L556–L562. doi:10.1152/ajplung.00360.2001. 11839552

[30] Salathe M, Lipson EJ, Ivonnet PI, Bookman RJ. 1997. Muscarinic signaling in ciliated tracheal epithelial cells: dual effects on Ca2+ and ciliary beating. Am J Physiol 272:L301–L310. doi:10.1152/ajplung.1997.272.2.L301. 9124382

[31] Jiao J, Zhang L. 2019. Influence of intranasal drugs on human nasal mucociliary clearance and ciliary beat frequency. Allergy Asthma Immunol Res 11:306–319. doi:10.4168/aair.2019.11.3.306. 30912321 PMC6439188

[32] Lazarowski ER, Boucher RC. 2009. Purinergic receptors in airway epithelia. Curr Opin Pharmacol 9:262–267. doi:10.1016/j.coph.2009.02.004. 19285919 PMC2692813

[33] Li Bassi G, Luque N, Martí JD, Aguilera Xiol E, Di Pasquale M, Giunta V, Comaru T, Rigol M, Terraneo S, De Rosa F, Rinaudo M, Crisafulli E, Peralta Lepe RC, Agusti C, Lucena C, Ferrer M, Fernández L, Torres A. 2015. Endotracheal tubes for critically ill patients: an in vivo analysis of associated tracheal injury, mucociliary clearance, and sealing efficacy. Chest 147:1327–1335. doi:10.1378/chest.14-1438. 25500677

[34] Li Bassi G, Zanella A, Cressoni M, Stylianou M, Kolobow T. 2008. Following tracheal intubation, mucus flow is reversed in the semirecumbent position: possible role in the pathogenesis of ventilator-associated pneumonia. Crit Care Med 36:518–525. doi:10.1097/01.CCM.0000299741.32078.E9. 18176317

[35] Nakagawa NK, Franchini ML, Driusso P, de Oliveira LR, Saldiva PHN, Lorenzi-Filho G. 2005. Mucociliary clearance is impaired in acutely ill patients. Chest 128:2772–2777. doi:10.1378/chest.128.4.2772. 16236954

[36] Germaud P, Haloun A. 2001. Invasive nosocomial pulmonary aspergillosis. Rev Pneumol Clin 57:157–163. 11353922

[37] De Pascale G, Tumbarello M. 2015. Fungal infections in the ICU: advances in treatment and diagnosis. Curr Opin Crit Care 21:421–429. doi:10.1097/MCC.0000000000000230. 26165502

[38] Fernández-Ruiz M, Puig-Asensio M, Guinea J, Almirante B, Padilla B, Almela M, Díaz-Martín A, Rodríguez-Baño J, Cuenca-Estrella M, Aguado JM, REIPI. 2015. Candida tropicalis bloodstream infection: incidence, risk factors and outcome in a population-based surveillance. J Infect 71:385–394. doi:10.1016/j.jinf.2015.05.009. 26033696

[39] Candoni A, Caira M, Cesaro S, Busca A, Giacchino M, Fanci R, Delia M, Nosari A, Bonini A, Cattaneo C, Melillo L, Caramatti C, Milone G, Scime' R, Picardi M, Fanin R, Pagano L, SEIFEM Group (Sorveglianza Epidemiologica Infezioni Fungine nelle Emopatie Maligne). 2014. Multicentre surveillance study on feasibility, safety and efficacy of antifungal combination therapy for proven or probable invasive fungal diseases in haematological patients: the SEIFEM real-life combo study. Mycoses 57:342–350. doi:10.1111/myc.12161. 24373120

[40] Chmiel JF, Aksamit TR, Chotirmall SH, Dasenbrook EC, Elborn JS, LiPuma JJ, Ranganathan SC, Waters VJ, Ratjen FA. 2014. Antibiotic management of lung infections in cystic fibrosis. II. Nontuberculous mycobacteria, anaerobic bacteria, and fungi. Ann Am Thorac Soc 11:1298–1306. doi:10.1513/AnnalsATS.201405-203AS. 25167882 PMC5469357

[41] Mellinghoff SC, Cornely OA, Jung N. 2018. Essentials in Candida bloodstream infection. Infection 46:897–899. doi:10.1007/s15010-018-1218-1. 30218311

[42] Chandrasekar P, Sirohi B, Seibel NL, Hsu JW, Azie N, Wu C, Ruhnke M. 2018. Efficacy of micafungin for the treatment of invasive candidiasis and candidaemia in patients with neutropenia. Mycoses 61:331–336. doi:10.1111/myc.12748. 29364548

[43] Ou HT, Lee TY, Chen YC, Charbonneau C. 2017. Pharmacoeconomic analysis of antifungal therapy for primary treatment of invasive candidiasis caused by Candida albicans and non-albicans Candida species. BMC Infect Dis 17:481. doi:10.1186/s12879-017-2573-8. 28693479 PMC5504557

[44] Castagnola E, Moresco L, Cappelli B, Cuzzubbo D, Moroni C, Lanino E, Faraci M. 2007. Nebulized liposomal amphotericin B and combined systemic antifungal therapy for the treatment of severe pulmonary aspergillosis after allogeneic hematopoietic stem cell transplant for a fatal mitochondrial disorder. J Chemother 19:339–342. doi:10.1179/joc.2007.19.3.339. 17594932

[45] Zhang J, Wang X, Vikash V, Ye Q, Wu D, Liu Y, Dong W. 2016. ROS and ROS-mediated cellular signaling. Oxid Med Cell Longev 2016:4350965. doi:10.1155/2016/4350965. 26998193 PMC4779832

[46] Bánsághi S, Golenár T, Madesh M, Csordás G, RamachandraRao S, Sharma K, Yule DI, Joseph SK, Hajnóczky G. 2014. Isoform- and species-specific control of inositol 1,4,5-trisphosphate (IP3) receptors by reactive oxygen species. J Biol Chem 289:8170–8181. doi:10.1074/jbc.M113.504159. 24469450 PMC3961646

[47] Booth DM, Enyedi B, Geiszt M, Varnai P, Hajnoczky G. 2016. Redox nanodomains are induced by and control calcium signaling at the ER-mitochondrial interface. Mol Cell 63:240–248. doi:10.1016/j.molcel.2016.05.040. 27397688 PMC4998968

[48] Sena LA, Chandel NS. 2012. Physiological roles of mitochondrial reactive oxygen species. Mol Cell 48:158–167. doi:10.1016/j.molcel.2012.09.025. 23102266 PMC3484374

[49] Houtmeyers E, Gosselink R, Gayan-Ramirez G, Decramer M. 1999. Effects of drugs on mucus clearance. Eur Respir J 14:452–467. doi:10.1034/j.1399-3003.1999.14b35.x. 10515429

[50] Houtmeyers E, Gosselink R, Gayan-Ramirez G, Decramer M. 1999. Regulation of mucociliary clearance in health and disease. Eur Respir J 13:1177–1188. doi:10.1034/j.1399-3003.1999.13e39.x. 10414423

[51] Weiterer S, Kohlen T, Veit F, Sachs L, Uhle F, Lichtenstern C, Weigand MA, Henrich M. 2015. Galactomannan and zymosan block the epinephrine-induced particle transport in tracheal epithelium. PLoS One 10:e0143163-14. doi:10.1371/journal.pone.0143163. 26571499 PMC4646458

[52] Pappas PG, Kauffman CA, Andes D, Benjamin DK, Calandra TF, Edwards JE, Filler SG, Fisher JF, Kullberg B-J, Ostrosky-Zeichner L, Reboli AC, Rex JH, Walsh TJ, Sobel JD, Infectious Diseases Society of America. 2009. Clinical practice guidelines for the management of candidiasis: 2009 update by the Infectious Diseases Society of America. Clin Infect Dis 48:503–535. doi:10.1086/596757. 19191635 PMC7294538

[53] Cornely OA, Cuenca-Estrella M, Meis JF, Ullmann AJ. 2014. European Society of Clinical Microbiology and Infectious Diseases (ESCMID) Fungal Infection Study Group (EFISG) and European Confederation of Medical Mycology (ECMM) 2013 joint guidelines on diagnosis and management of rare and emerging fungal diseases. Clin Microbiol Infect 20(Suppl 3):1–4. doi:10.1111/1469-0691.12569. 24606200

[54] Tamaoki J, Chiyotani A, Kondo M, Konno K. 1995. Role of NO generation in beta-adrenoceptor-mediated stimulation of rabbit airway ciliary motility. Am J Physiol 268:C1342–C1347. doi:10.1152/ajpcell.1995.268.6.C1342. 7611351

[55] Uzlaner N, Priel Z. 1999. Interplay between the NO pathway and elevated [Ca^2+^]_i_ enhances ciliary activity in rabbit trachea. J Physiol 516:179–190. doi:10.1111/j.1469-7793.1999.179aa.x. 10066932 PMC2269217

[56] Li D, Shirakami G, Zhan X, Johns RA. 2000. Regulation of ciliary beat frequency by the nitric oxide-cyclic guanosine monophosphate signaling pathway in rat airway epithelial cells. Am J Respir Cell Mol Biol 23:175–181. doi:10.1165/ajrcmb.23.2.4022. 10919983

[57] Jiao J, Wang H, Lou W, Jin S, Fan E, Li Y, Han D, Zhang L. 2011. Regulation of ciliary beat frequency by the nitric oxide signaling pathway in mouse nasal and tracheal epithelial cells. Exp Cell Res 317:2548–2553. doi:10.1016/j.yexcr.2011.07.007. 21787770

[58] Kobayashi K, Salathé M, Pratt MM, Cartagena NJ, Soloni F, Seybold ZV, Wanner A. 1992. Mechanism of hydrogen peroxide-induced inhibition of sheep airway cilia. Am J Respir Cell Mol Biol 6:667–673. doi:10.1165/ajrcmb/6.6.667. 1591015

[59] Kakuta Y, Kanno T, Sasaki H, Takishima T. 1985. Effect of Ca^2+^ on the ciliary beat frequency of skinned dog tracheal epithelium. Respir Physiol 60:9–19. doi:10.1016/0034-5687(85)90036-2. 3873667

[60] Lansley AB, Sanderson MJ, Dirksen ER. 1992. Control of the beat cycle of respiratory tract cilia by Ca^2+^ and cAMP. Am J Physiol 263:L232–L242. doi:10.1152/ajplung.1992.263.2.L232. 1325130

[61] Varnai P, Hunyady L, Balla T. 2009. STIM and Orai: the long-awaited constituents of store-operated calcium entry. Trends Pharmacol Sci 30:118–128. doi:10.1016/j.tips.2008.11.005. 19187978 PMC3125588

[62] Fahrner M, Schindl R, Muik M, Derler I, Romanin C. 2017. The STIM-Orai pathway: the interactions between STIM and Orai. Adv Exp Med Biol 993:59–81. doi:10.1007/978-3-319-57732-6_4. 28900909

[63] Samanta K, Bakowski D, Parekh AB. 2014. Key role for store-operated Ca^2+^ channels in activating gene expression in human airway bronchial epithelial cells. PLoS One 9:e105586. doi:10.1371/journal.pone.0105586. 25157492 PMC4144895

[64] Prakriya M, Lewis RS. 2015. Store-operated calcium channels. Physiol Rev 95:1383–1436. doi:10.1152/physrev.00020.2014. 26400989 PMC4600950

[65] Alenmyr L, Uller L, Greiff L, Hogestatt ED, Zygmunt PM. 2014. TRPV4-mediated calcium influx and ciliary activity in human native airway epithelial cells. Basic Clin Pharmacol Toxicol 114:210–216. doi:10.1111/bcpt.12135. 24034343

[66] Rosenbaum T, et al. 2020. TRPV4: a physio and pathophysiologically significant ion channel. Int J Mol Sci 21:3837. doi:10.3390/ijms21113837. PMC7312103 32481620

[67] Lorenzo IM, Liedtke W, Sanderson MJ, Valverde MA. 2008. TRPV4 channel participates in receptor-operated calcium entry and ciliary beat frequency regulation in mouse airway epithelial cells. Proc Natl Acad Sci USA 105:12611–12616. doi:10.1073/pnas.0803970105. 18719094 PMC2527959

[68] Alpizar YA, Boonen B, Sanchez A, Jung C, López-Requena A, Naert R, Steelant B, Luyts K, Plata C, De Vooght V, Vanoirbeek JAJ, Meseguer VM, Voets T, Alvarez JL, Hellings PW, Hoet PHM, Nemery B, Valverde MA, Talavera K. 2017. TRPV4 activation triggers protective responses to bacterial lipopolysaccharides in airway epithelial cells. Nat Commun 8:1059. doi:10.1038/s41467-017-01201-3. 29057902 PMC5651912

[69] White JPM, Cibelli M, Urban L, Nilius B, McGeown JG, Nagy I. 2016. TRPV4: molecular conductor of a diverse orchestra. Physiol Rev 96:911–973. doi:10.1152/physrev.00016.2015. 27252279

[70] Müller S, Koch C, Weiterer S, Weigand MA, Sander M, Henrich M. 2020. Caspofungin induces the release of Ca(2+) ions from internal stores by activating ryanodine receptor-dependent pathways in human tracheal epithelial cells. Sci Rep 10:11723. doi:10.1038/s41598-020-68626-7. 32678179 PMC7367263

