# Supplementary material for: Echinocandins Accelerate Particle Transport Velocity in the Murine Tracheal Epithelium: Dependency on Intracellular Ca2+ Stores
Source: Antimicrob Agents Chemother. 2021 Oct 18;65(11):e00669-21. doi: 10.1128/AAC.00669-21 (PMC8522769; doi:10.1128/AAC.00669-21)
Supplement: Supplemental file 1 — Supplemental material. Download AAC.00669-21-s0001.pdf, PDF file, 0.3 MB [file aac.00669-21-s0001.pdf]

Supplement Fig. 1

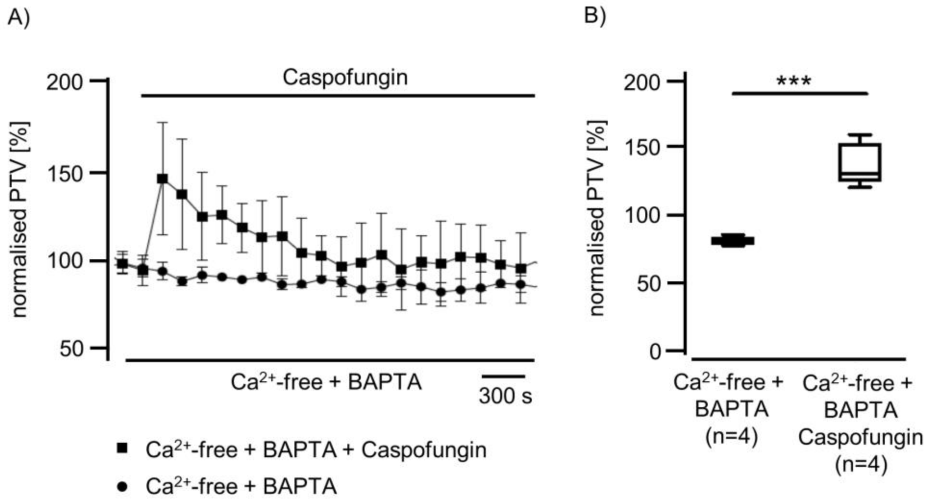

Chelating of intracellular Ca^{2+} ions reduces the duration of Caspofungin evoked rise in PTV.

A) In Ca^{2+}–free buffer medium tracheae were treated with BAPTA-AM to reduce free intracellular Ca^{2+} ions. The subsequent application of Caspofungin induced a significant transient rise of the PTV. Still the amplitude of the Caspofungin induces rise in PTV was significant, however this effect was transient, the PTV slowly decayed back to baseline during the exposure period. **B)** During application of BAPTA-AM the amplitude of the Caspofungin induced rise in PTV was significantly higher than the PTV under control conditions. (time scale bar: 300 sec., horizontal bars in experimental recording present exposure persiods, n = number of individual experiments ***$p < 0.001$, Mann–Whitney U test).

Supplement Fig. 2

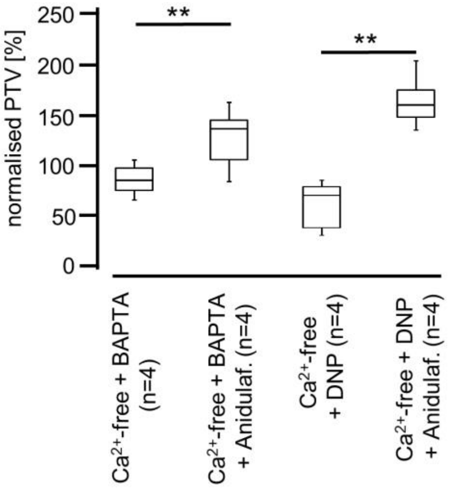

Anidulafungin evokes brief rise in PTV in trachea treated with BAPTA-AM or DNP.

Anidulafungin induced a significant transient rise in the PTV in Ca^{2+}-free medium supplemented with BAPTA-AM to minimize intracellular free Ca^{2+} ions. Application of DNP that uncouples the mitochondrial respiratory chain and depletes mitochondrial Ca^{2+} stores did not prevent the Anidulafungin-induced rise of the PTV. (n = number of individual experiments **$p < 0.01$, Mann–Whitney U test).
